# Supplementary material for: Deetect: A Deep Learning-Based Image Analysis Tool for Quantification of Adherent Cell Populations on Oxygenator Membranes after Extracorporeal Membrane Oxygenation Therapy
Source: Biomolecules. 2022 Dec 3;12(12):1810. doi: 10.3390/biom12121810 (PMC9776364; doi:10.3390/biom12121810)
Supplement: Supplementary file 1 [file biomolecules-12-01810-s001.zip › Supplementary Figure S1.pdf]

## Supplementary Figure S1:

In the study, samples were taken from different parts of the oxygenator along the direction of blood flow to get indications of possible differences in cell colonization. For this purpose, predilection sites in the venous, middle, and arterial parts were examined. The image sequences were processed by Deetect using 2-Z stacking and then compared (supplementary figure 1). The highest average cell count was found on the warps of the middle oxygenator section (657 ± 192) and the lowest on the hollow fibers of the venous part (264 ± 126). Thus, a trend was noted for increased cell colonization from the venous to the middle and arterial side of the oxygenator. The results of the Kruskal-Wallis and Dunn's tests indicate that there are significant differences between the cell deposition on predilection sites of hollow fibers and warps in different parts of the oxygenator.

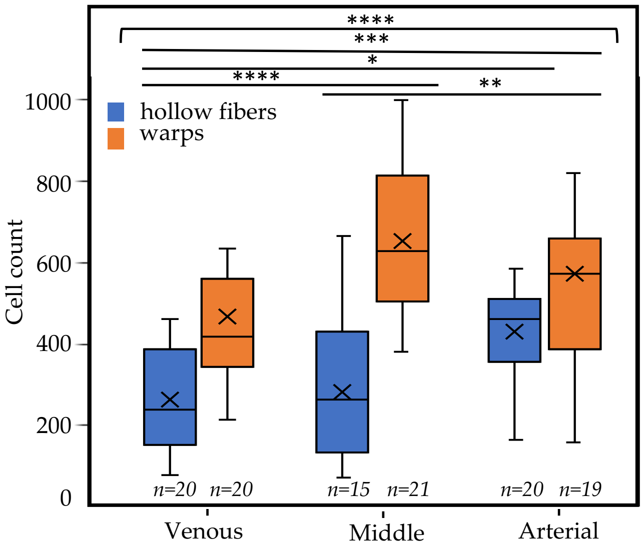


**Supplementary Figure S1.** Boxplot showing comparison of the total cell count of predilection sites in different parts of the oxygenator. Middle line=median; X=mean; box=interquartile range (IQR); upper/lower whisker (OW/UW)≈1.5xIQR. The brackets indicate the results of the Kruskal-Wallis-test. Result of the post hoc Dunn's test is only shown for differences between different parts of the MO (lines). The p-value is indicated using the following annotation: Ns, p≥0.05; *, p≤0.05; **, p≤0.01; ***, p≤0.001; ****, p≤0.0001.
